# A Standard Protocol for the Production and Bioevaluation of Ethical *In Vivo* Models of HPV-Negative Head and Neck Squamous Cell Carcinoma

**DOI:** 10.1021/acsptsci.1c00083

**Published:** 2021-04-14

**Authors:** Patrizia Sarogni, Ana Katrina Mapanao, Sabrina Marchetti, Claudia Kusmic, Valerio Voliani

**Affiliations:** † Center for Nanotechnology Innovation@NEST, Istituto Italiano di Tecnologia, Piazza San Silvestro 12, Pisa 56126, Italy; ‡ NEST-Scuola Normale Superiore, Piazza San Silvestro 12, Pisa 56126, Italy; § Institute of Clinical Physiology, CNR, Via G. Moruzzi 1, Pisa 56100, Italy

**Keywords:** chick chorioallantoic membrane, head/neck, cancer, alternative models, 3Rs principle, ethics

## Abstract

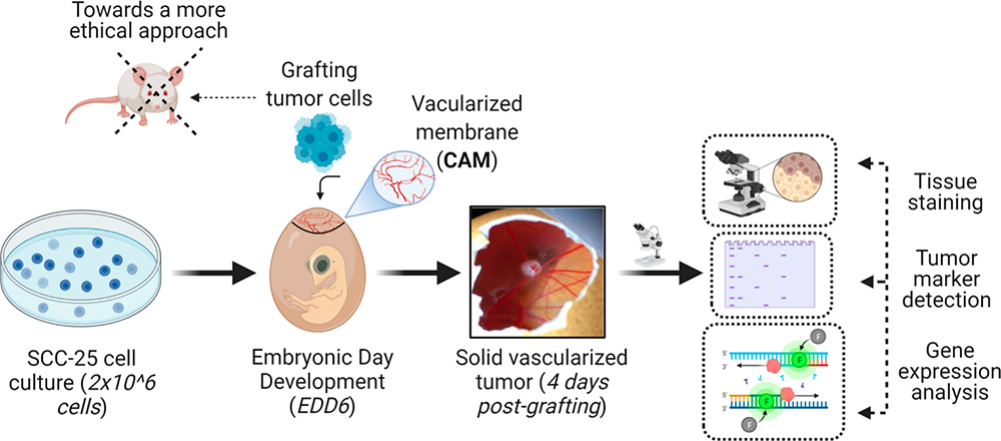

Preclinical cancer research increasingly demands sophisticated models for the development and translation of efficient and safe cancer treatments to clinical practice. In this regard, tumor-grafted chorioallantoic membrane (CAM) models are biological platforms that account for the dynamic roles of the tumor microenvironment and cancer physiopathology, allowing straightforward investigations in agreement to the 3Rs concept (the concept of reduction, refinement, and replacement of animal models). CAM models are the next advanced model for tumor biological explorations as well as for reliable assessment regarding initial efficacy, toxicity, and systemic biokinetics of conventional and emerging neoplasm treatment modalities. Here we report a standardized and optimized protocol for the production and biocharacterization of human papillomavirus (HPV)-negative head and neck chick chorioallantoic membrane models from a commercial cell line (SCC-25). Oral malignancies continue to have severe morbidity with less than 50% long-term survival despite the advancement in the available therapies. Thus, there is a persisting demand for new management approaches to establish more efficient strategies toward their treatment. Remarkably, the inclusion of CAM models in the preclinical research workflow is crucial to ethically foster both the basic and translational oncological research on oral malignancies as well as for the advancement of efficient cancer treatment approaches.

Head and neck squamous cell carcinomas (HNSCCs) represent a wide class of epithelial neoplasms localized in the oral and nasal cavities, paranasal sinuses, salivary glands, pharynx, and larynx and whose molecular mechanisms involved in the progression of the disease are still to be completely clarified.^1,2^ One of the leading causes of the development of HNSCCs is the long-term consumption of tobacco and alcohol.^3^ Another associated risk factor for the onset of HNSCCs is human papillomavirus (HPV) infection.^4^ The presence of HPV usually affects the prognosis of the disease, with more favorable outcomes for the patients.^4,5^ The general workup for staging and diagnosis of squamous cell carcinoma of the oral cavity, larynx, oropharynx, and hypopharynx includes physical examinations, medical history, blood test, MRI/CT imaging, and biopsy under local anesthesia.^4^ These features together with the evaluations on the genomic alterations or gene expression profile are important for the pathological staging and prognosis and to determine the type of treatment. In general, the usual management strategies for oral cavity, laryngeal, oropharyngeal, and hypopharyngeal cancers are different between locally advanced tumors and early stage tumors.^6^ Indeed, according with the most recent Clinical Practice Guidelines in Oncology (e.g., NCCN), the standard treatments in early stage disease, consisting of conservative surgery or radiotherapy, give similar locoregional control. Standard options for locally advanced HNSCC are either surgery plus adjuvant chemo/radiotherapy or primary chemoradiotherapy alone, even if the best treatment regime is chosen on a case-by-case analysis.^4,7^ In the field of prognostic estimation, it appears that cases of HNSCC that survive beyond 5 years after the initial diagnosis show decreased overall survival when compared to noncancer subjects of the same age.^8^ In general, stage I cases had improved survival compared to stage II–IV, where no particular difference was proved in long-term survival for cases alive 5 years after diagnosis. However, site, stage, smoking, and cardiovascular disease are significant factors determinant of mortality.^8^ Nevertheless, oral malignancies, including tongue cancer, continue to have severe morbidity with less than 50% long-term survival despite the advancement in the available and emerging therapies.^4,5,9,10^ In this regard, understanding the biological processes at the basis of oral malignancies and the development of new therapeutic strategies to improve the survival rate of patients while preserving the structure and function of the involved organs are crucial topics for their management.^11^ In this context, *in vivo* models are pivotal to foster the advancements in oncology by bridging the gap between preclinical investigations and human clinical trial on conventional and emerging therapeutic approaches as well as to understand tumor cell behaviors in a physiological environment.^16^ The most widely employed *in vivo* models are murine, and they have been crucial to establish most of the current models in pediatric oncology and to develop some drugs, among which topotecan and irinotecan.^7^ Moreover, murine models are particularly relevant for absorption–distribution–metabolism–excretion–toxicity (ADMET) investigations.^113−115^ However, genetically immunocompromised murine models have very high costs of maintenance, and the tumor engraftment may require up to 4 months. Engraftment failure can be high and cannot be usually determined before some months post-implantation, and their employment is increasingly discouraged by following the 3Rs concept (the concept of reduction, refinement, and replacement of animal models) and the European Parliament Directive 2010/63/EU.^13,14^ Among other biological models, the chick chorioallantoic membrane (CAM) is one of the most attractive and ethical *in vivo* models that jointly combines reliability, medium-/high-throughput screenings, and easy handling during imaging/treatment evaluations.^15−16^ Notably, the absence of a mature immune system of the embryo during the early developmental stage reduces the risk of tumor rejection after implantation.^16^ Thus, CAM models can develop visible solid tumors within 4–5 days after engraftment, in comparison to the approximately 3–6 weeks of murine models.^7^ Besides the usually high rate of success for tumor grafting, other major advantages of CAM models are the easy daily inspection of the tumor site and their flexibility of employment. Moreover, CAM models are ethical models in agreement with the 3Rs concept because the chick embryo does not develop pain perception before the 17th day of incubation.^16^ Indeed, investigations in CAM models usually do not require permissions or approval from ethics committees.^16^ Despite some difficulties in the application of CAM models to long-term investigations, their highly vascularized membrane together with the immature immune response allowed the low rejection rate grafting of several tissues and the study of various neoplasms, including osteosarcoma, glioblastoma, pancreatic carcinoma, and colon carcinoma.^17−20^ Several variable tumor engraftment methods for CAM model production have been reported, yet these studies focus on the tumor biology or the evaluation of a treatment avoiding reporting a standard protocol for the production of the models.^21,22^ In general, in the literature there is a serious lack of work regarding the standard production of CAM models. Thus, a detailed step-by-step procedure for the reliable CAM tumor model fabrication together with the standard biocharacterization is required to serve as guide for researchers interested to advance the field.

In order to address this demand, we report a standardized protocol for the composition as well as the cascade assays for the characterization of a commercial HPV-negative head and neck cell line (SCC-25) grafted on chick chorioallantoic membrane of fertilized Leghorn chicken eggs.

## Materials

### Reagents and Consumables

- Fertilized red or white Leghorn chicken eggs
- SCC-25 squamous cell carcinoma cell line (ATCC, catalog number CRL-1628)
- DMEM/Ham’s F12 1:1 medium (DMEM/F12 Gibco, 21041025)
- Fetal bovine serum, qualified, heat-inactivated (FBS, Thermo Fisher Scientific, 10500064)
- l-Glutamine (Thermo Fisher Scientific, A2916801)
- Hydrocortisone (Sigma-Aldrich, H0888)
- Penicillin–streptomycin (Pen/Strep 100X, 5,000 U/mL) (Thermo Fisher Scientific, 15070063)
- Phosphate-buffered saline without calcium and magnesium (PBS, Sigma-Aldrich, D8537)
- Trypsin-EDTA (0.5%), phenol red (Thermo Fisher Scientific, 25300054)
- Serological pipette, pipette tips, microcentrifuge tubes, conical tubes, and flasks
- TC Dish150, Standard (83.3903, SARSTEDT)
- Matrigel Matrix (Corning, ref 354234)
- Sterile water
- Fixative solution, i.e., 4% paraformaldehyde (PFA) in PBS
- RNA extraction kit, Nucleospin RNA plus (740984.50 MACHEREY-NAGEL)
- cDNA synthesis kit, iScript cDNA Synthesis (1708891 BIORAD)
- iTaqUniversal SYBRGreen Supermix (1725121 BIORAD)
- RIPA buffer (Pierce 89901)
- Protease Inhibitors Cockatil Tablets (04693116001 Roche)
- Bradford Reagent (B6916 Sigma-Aldrich)
- Albumin Standard (23209 Thermo scientific)
- Nitrocellulose membrane, TransBlot Turbo Midi-size nitrocellulose (1620167 BIORAD)
- anti-TFRC primary antibody (SAB4200398 Sigma-Aldrich)
- Goat anti-rabbit IgG (H+L)-HRP-conjugated secondary antibody, (170–6515 BIORAD)
- Clarity Western ECL substrate (1705061 BIORAD)
- Paraffin wax (melting point 56 °C)
- Ethanol (70, 80, 95, and 100% alcohol)
- Xylene
- Mayer’s hematoxylin solution
- Eosin Y aqueous solution 1%
- Permount mounting medium

### Equipment and Tools

- Cell counter (Invitrogen Countess cell counter)
- Optical microscope
- Egg incubator, 37.5 °C/99.5 °F, 60% humidity, FIEM MG 140/200
- Tilting egg racks
- Sterile dissection scissors and tweezers
- Soft tissue paper or cotton swab
- Refrigerator or cold room at 4 °C
- Analytical balance
- Ruler
- Adhesive tapes, preferably Scotch magic tape
- Portable digital microscope, Dino-Lite AM7915MZT (or any camera-associated microscope)
- DinoCapture 2.0 Software
- Rotary microtome
- Forced ventilation histology oven
- Paraffin-embedding station
- Light microscope equipped with RGB video camera

## Methods

### Experimental Outline

**Figure 1:**
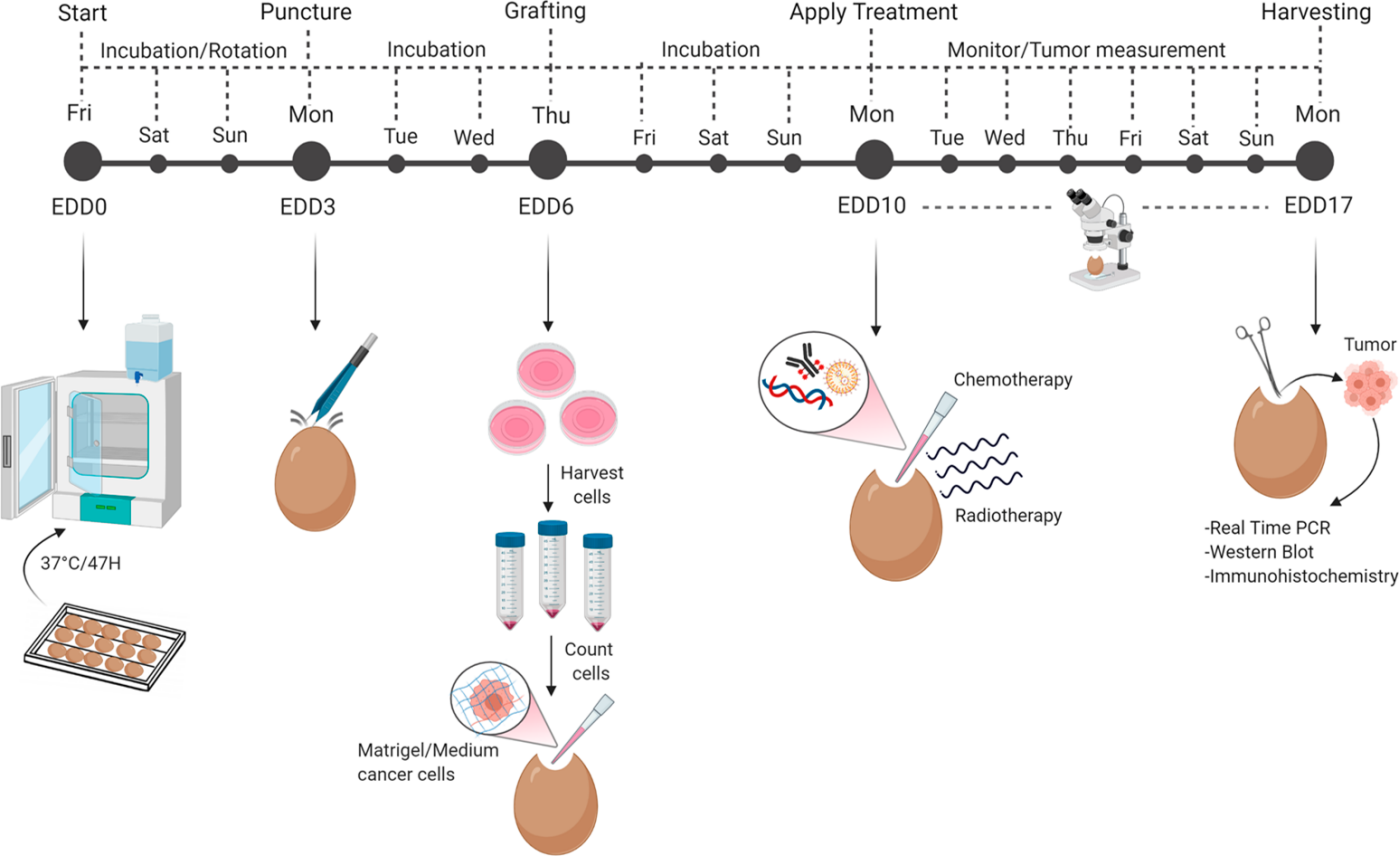
Overview of the CAM schedule from EDD0 to EDD17. In general, chick embryo incubation is marked as embryonic day of development 0 (EDD0). The puncturing day (EDD3) allows the translocation of the natural air sac to the top of the egg. Grafting of 2 × 10^6^ SCC-25 cells (EDD6) enables the generation of a solid visible tumor at 4 days post-grafting (EDD10). The topical treatment is applied on EDD10, and the tumor mass is monitored until EDD17, the last day of incubation (harvesting).

The establishment of the experimental design is the first important step to define a complete outline of the assay procedure. For the optimization of the CAM assay employing SCC-25 cells, the schedules of egg incubation and subsequent experimentations were based on the proposed scheme by Kleibeuker et al. (Figure 1).^23^ In general, the start of the incubation corresponds to the embryonic day of development 0 (EDD0). Starting from this moment, under appropriate environmental conditions, the fertilized eggs begin their embryonic growth. It is important to take into account that the biological window available to perform this assay must not exceed the 17 days of incubation to prevent the hatching of the eggs and avoid ethical restrictions.^16^

### Cleaning the Working Area and Eggs (Start of Incubation)

**Figure 2:**
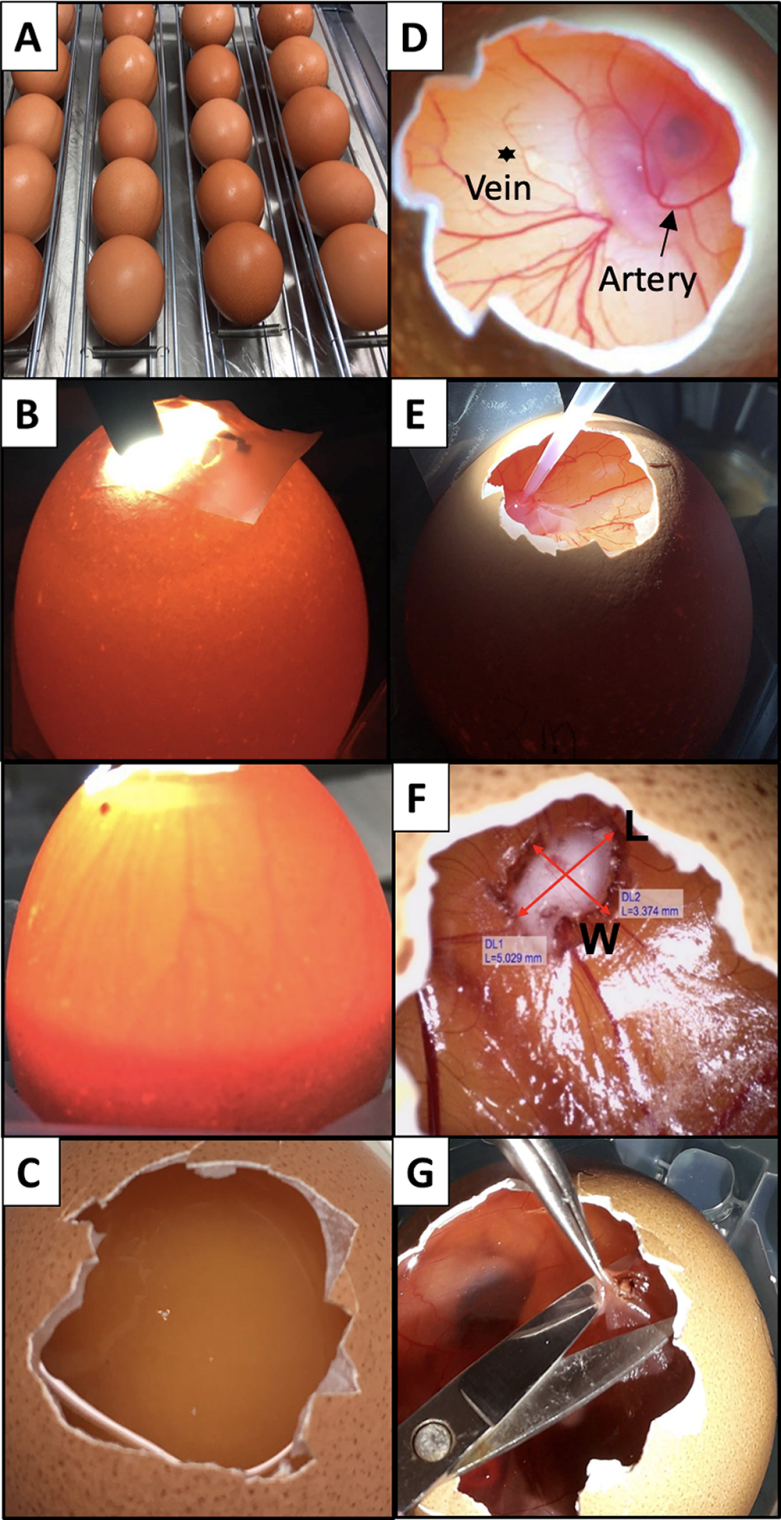
Images of the main steps of the CAM protocol. (A) Eggs are horizontally placed in the tilting trays before the incubation starts. (B) A light source placed on top/behind the egg allows identification of the location of the air chamber and the existence of a vascular network (Top, infertile egg; bottom, fertile egg). (C) An unfertilized egg is associated with the absence of the embryo and of the vascular network. (D) Distinction between artery (arrow) and vein (asterisk). Arteries are thicker and darker compared to veins.^25^ (E) Image showing the grafting procedure of SCC-25 cancer cells onto the bleeding blood vessel. (F) Tumor volume measurement determined by means of width and length. The length is associated with the longest diameter of the tumor mass. (G) Harvesting procedure of the tumor at EDD17. The CAM membrane is gently lifted with tweezers and the tumor is cut with scissors.

It is highly recommended to work under sterile conditions and to adequately clean the working area with 70% ethanol and/or bleach to avoid any type of microorganism contamination. Carefully clean the shell of each egg with soft tissue paper soaked in distilled water before fitting them into the tray.^230^ Place the eggs horizontally next to each other and insert a steel spring in the ends of the columns to secure the eggs while the racks tilt (Figure 2A).^231^ Make sure that the temperature and humidity of the incubator reach the appropriate parameters before starting the incubation (37.5 °C, ∼47% humidity).^232^

### Puncturing

Take the eggs out after 3 days of incubation (EDD3) and put them in upright position so that the air sac will translocate to the top of the eggs.^233^ Gently scratch with tweezers the tip of each egg until the shell becomes fragile and breakable, finally making a small hole. Seal the hole with an adhesive tape to prevent dehydration and infection, and put the eggs back in the incubator in the stationary mode (no tilting).

### Tumor Grafting

#### Selection of Eggs

At EDD6, the fertilized eggs must be accurately selected before proceeding with the grafting of the tumor cell suspension on the CAM. Remove the eggshell around the small hole to create a small window of about 1 cm^2^.^234^ Check the fertilization and discard the unfertilized eggs (Figure 2C). Label the eggs with the appropriate information (e.g., egg number, cell density, name of the cell line, and treatment conditions) to avoid confusion. Reseal the window, and put the eggs back in the incubator.

#### Preparation of Tumor Cell Suspension

Harvest the SCC-25 cells, and collect them in one 50 mL tube.^235^ Spin down the cells, and discard the supernatant. Resuspend the pellet in fresh cell culture medium. Count the cells, and adjust the concentration in order to have at least 2 × 10^6^ cells per egg.^236^ Spin down the cells again, and discard the supernatant. Resuspend the pellet in a mixture of Matrigel/medium without FBS (in ratio 1:1) such that each egg will be dispensed with 25 μL of the cell suspension.^237^

#### Grafting

Open the sealed window. Roll a soft tissue paper and gently poke a small vein within the CAM region until it starts to bleed (Figure 2D).^238^ Pipette 25 μL of the cell suspension on top of the bleeding blood vessel. Reseal the window, and put the eggs in the incubator (Figure 2E). Always make sure that the humidifier and the tank are filled with water and that the incubator is still maintaining the right conditions of temperature and humidity.

### Apply Treatment (EDD10)

A reference concentration of your drug/chemotherapeutic compound to be tested is recommended.^239^ The amount of the treatment solution to be prepared will depend on the number of tumor-grafted eggs to be treated, while the frequency of the treatment will depend on your own schedule and experimental objectives. To proceed, take the eggs out from the incubator, and mark each egg for the corresponding treatment to avoid confusion. For our experiments, each egg is topically administered with chemotherapeutic drugs/test compounds suspended in 30 μL of solution (serum-free medium).^240^

### Tumor Growth Monitoring (EDD10–EDD17)

Place a ruler under the DinoLite camera and calibrate the software before starting to acquire images of the tumors. Although constantly noting the calibration by using the ruler can help in obtaining consistent and accurate measurements, it is also advised to take photographs at the same magnification to better visualize the changes in tumor dimensions. The complementary DinoLite software (DinoCapture 2.0) comes with length measurement tools. Measure the tumor sizes, in which the longer and shorter measurements are denoted as the length (*L*) and width (*W*), respectively (Figure 2F). From these measurements, the tumor volume is derived using a modified ellipsoid formula: 0.5(*L* × (*W*^2^)).^241,19^

### Tumor Harvesting (EDD17)

On EDD17, take the eggs out of the incubator and place them in the cold room for at least 2 h to restrict the movements of the chick embryo. Thoroughly clean the dissection area, tweezers, and scissors with 70% ethanol and bleach. Prepare wash containers with ethanol and PBS for rinsing the tools to avoid cross-contamination among different tumors. Open the sealed window, and remove some eggshell around to make the tumor more accessible for cutting. Carefully lift the membrane with tweezers. Cut the tumor, and place it in a Petri dish with PBS. Take a photograph of the tumor, and place it in an empty microcentrifuge tube, and weigh it (Figure 2G).^242^ Store the harvested tumor samples directly in −80 °C or in formalin fixing solution for further analysis.

## Downstream Assays

### Quantitative Real Time-PCR

To extract the total RNA from the SCC-25 cell line, we used Nucleospin RNA plus Kit (740984.50 MACHEREY-NAGEL) following the manufacturer’s instruction. Briefly, the harvested tissue is minced in small pieces using a plastic pestle. The extracted RNA can be used immediately or stored at −80 °C. The quality control of RNA through agarose gel electrophoresis will avoid the likelihood of final problem solving, such as in cDNA reverse transcription and amplification of the gene target. For cDNA synthesis, 500 ng of RNA was reverse-transcribed with iScript cDNA Synthesis Kit (1708891 BIORAD). Dilute 500 ng of the total cDNA 1:10 in nuclease-free water in order to get 50 ng as final amount before using it for the PCR reaction. Quantitative real-time PCR is carried out using iTaqUniversal SYBRGreen Supermix (1725121 BIORAD). To prepare the PCR reaction mix in a final volume of 20 μL, use 1–2 μL of the diluted cDNA with around 5–10 ng of cDNA template. Use specific primers for your gene of interest and primers for a housekeeping gene. One of the commonly utilized housekeeping genes, which we also use, is GAPDH. All the samples are prepared in triplicate. The amplification curves are visualized by SYBR Green Analysis on Applied Biosystem Instrument (7300). The recommended thermal cycling for the amplification is as follows: 95 °C for 10 min, 40 cycles at 95 °C 15 s, 64 °C for 30 s, and 72 °C for 30 s. The 2^–ΔΔCT^ method is used to calculate the relative expression level.^24^

### Western Blotting

Cells pellets are resuspended in RIPA buffer (Pierce 89901) supplemented with protease inhibitors and mechanically minced using a 200 μL pipet tip. The lysates are then incubated for 30 min in ice, and the supernatants are collected after centrifugation for 30 min at 14 000 rpm. The protein concentration in the lysates is determined through the Bradford assay, using a standard calibration curve method prepared with bovine serum albumin (BSA) of known concentrations (2000 μg/mL). The absorbance values at 595 nm of the samples and the standards are noted. The formula *y* = *mx* + *q* is used to derive the protein concentration. Then, 30–50 μg of the total protein was separated via SDS-PAGE. Upon transferring the samples from the gel to a nitrocellulose membrane, the proteins are treated with a blocking solution (TBS 1× 5% powdered milk) for 1 h at room temperature. For the overnight primary antibody incubation at 4 °C, we used anti-TFRC primary antibody (SAB4200398 Sigma-Aldrich). The membrane was washed thrice with TBS 1×–0.1% Tween20, and the horseradish peroxidase (HRP)-conjugated secondary antibody was then added and incubated for 1 h at room temperature. Finally, after further washing in TBS 1×–0.1% Tween20, the bands are visualized through chemiluminescence using an enhanced chemiluminescence (ECL) kit (1705061 Biorad) and Image Quant LAS 4000 System.

### Hematoxylin and Eosin (H&E) Staining

Tumor samples, fixed in 4% paraformaldehyde for 24–48 h, were rinsed in running tap water for 10–15 min. Then, dehydrate samples through increasing alcohol series, followed by three changes of 100% alcohol applied for 5 min each. The tissues were cleared in xylene for 12 min and then immersed in paraffin wax for 10 min, followed by other two paraffin changes, 5 min each, and finally embedded in paraffin blocks. Serial sections of 5–6 μm were cut with a microtome, placed on slides, and heated overnight at 40 °C in forced ventilation histology oven. The sections were cleared from paraffin by two changes in xylene, 6 min each. The tissue was hydrated through decreasing ethanol series and rinsed in distilled water for at least 5 min.

Slices were stained with Mayer’s hematoxylin solution for 5 min, followed by a 10 min rinse in running tap water, and finally stained in eosin Y aqueous solution for 2 min. Slides were rinsed in distilled water, heated at 40 °C for 40 min, dipped twice in xylene, and a coverslip was added placing a drop of Permount mounting medium.

Histological images (40×, 100×, 200×, and 400×) are acquired by light microscope (Olympus BX43, Japan) and digitized using a RGB video camera (Olympus DP 20, Japan).

## Anticipated Results

**Figure 3:**
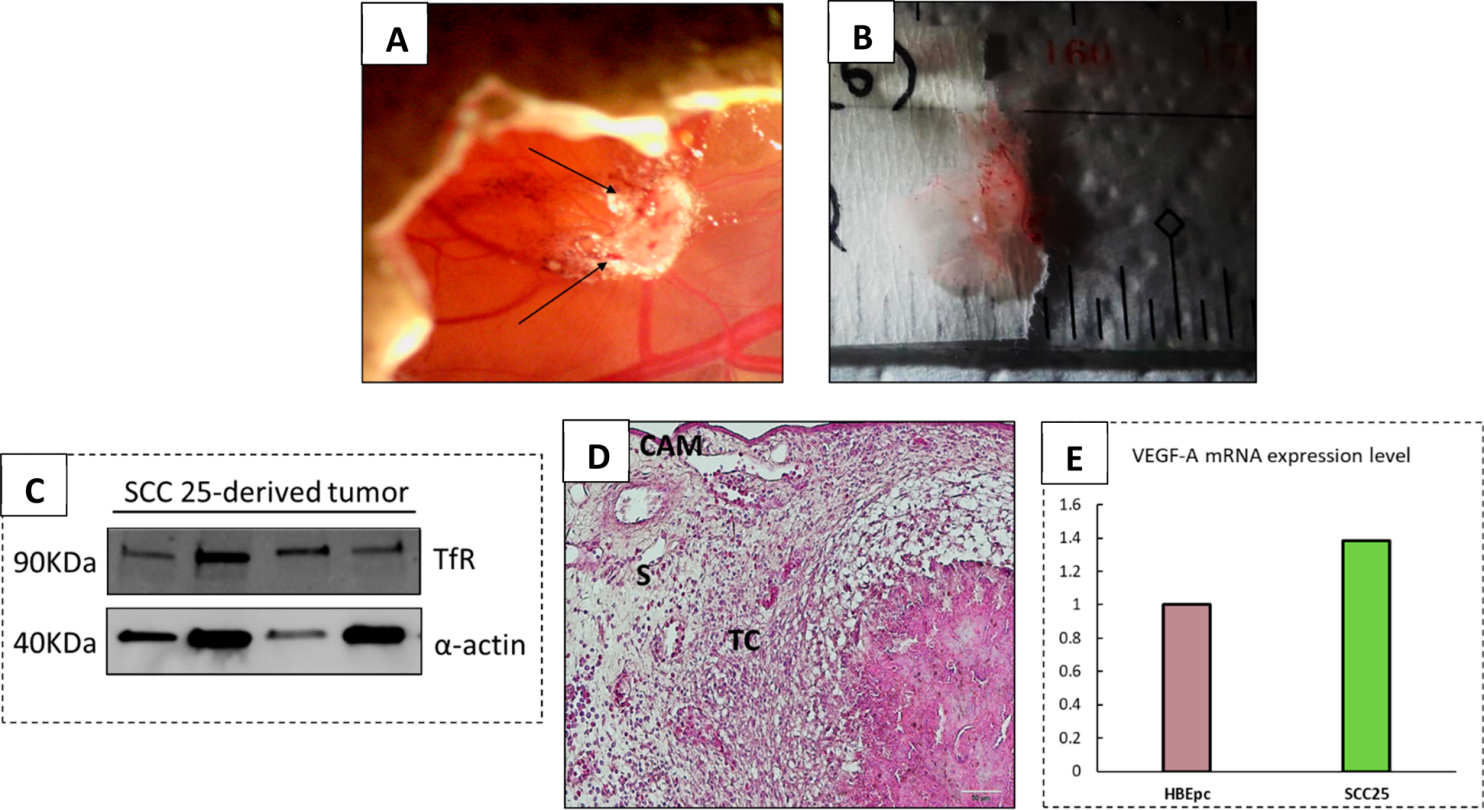
Characterization of harvested SCC25 tumors. (A) Representative image of SCC-25 solid tumor grown onto the CAM. The arrows indicate the blood vessels across the tumor mass. (B) Example of solid and vascularized tumor harvested at EDD17. (C) Western blotting analysis depicting the expression of the TfR marker in SCC-25-tumor-derived cells. (D) H&E staining of SCC-25 tumor-derived cells showing the tissue structure and cells distribution (TC = tumor cells; S = stroma; scale bar = 20 μm). (E) Real-time PCR measurement of VEGF-A mRNA expression levels in SCC-25 cancer cells compared to HBEpc bronchial cell line.

The reliable generation of a solid tumor on CAM is fundamental for both the comprehension of *in vivo* cancer cell behaviors and the efficacy/toxicity evaluation of emerging and conventional therapeutic strategies. Imaging of tumors provides detailed information about the quality and the presence/absence of blood vessel across the tumor itself; an important parameter to monitor during the time frame of the experiment and/or after the therapeutic treatments.^26^ Imaging also provides a practical method to identify the volume of the tumor mass without interfering with its spatial organization and allows for distinguishing the tumor from artifacts caused by the aggregation of the Matrigel solution. The primary goals in the production of CAM tumor models include the achievement of a high embryo survival rate and visible tumor grafting that allow the topical application of therapeutics. Our optimized protocol allows the development of solid vascularized SCC-25 tumor with high efficiency (∼80%) and with a volume of 5–20 mm^3^ at EDD10 (Figure 3A). It is important to notice that the assessment of tumor size following the harvesting can be affected by the wrinkling of the membrane after 2 h at 4 °C. However, the size of the excised tumors usually significantly correlates with their *in vivo* volume. Measuring the weight of the excised tumors is a facile and useful end-point evaluation that provides additional information on the aggressiveness and ability of cancer cells to grow and form solid tumors (Figure 3B). It is worth remembering that the identification and introduction of new molecular pathways involved in the neoplastic transition has rapidly expanded, considerably advancing the diagnostic techniques.^27^ In fact, the detection of specific biomolecular tumor markers represents a significant diagnostic screening approach that allows differentiation between cancer cells and the surrounding cells. Among the number of molecular mechanisms already identified in neoplasms, the expression of transferrin receptor (TfR) appears to be compromised in HNSCCs, leading to an overexpression of the receptor and therefore constituting a promising tumor marker.^28,29^ In this regard, to effectively prove the human origin of the harvested tissue, Western blot analysis has been carried out using an antihuman TfR antibody. The results demonstrate the presence of the TfR protein maker in the harvested tumor samples, confirming the significance of this approach to determinate the human origin of the grafted tumor (Figure 3C).

H&E staining is another pivotal end-point assay that mainly provides qualitative information and a general overview on the structure of the tissues.^30^ Remarkably, this approach enables visualization of cellular morphology and the distribution pattern of distinct cells, as well as their density and consistency. The H&E staining of the collected SCC-25 tumor specimen showed a well-organized, preserved, and homogeneous structure, clearly identifiable from the surrounding stroma and the membrane border (Figure 3D).

Since genetic alterations are often events upstream of cell metabolism dysfunction, advances in the assessment of gene expression profile may be useful for the prognosis of various type of cancers, hence offering a wide overview about the gene interaction network in the development of the disease. In fact, mutations that lead to the alteration of gene expression level of cytokines, enzymes, and growth factors are the main responsible of tumorigenesis.^31^ The deregulation of the vascular endothelial growth factor-A (VEGF-A) was observed to be among the tumor promoting factors in SCC-25 cells. Indeed, VEGF-A is relatively overexpressed in SCC-25 cells compared to the level in normal cells (Figure 3E) and constitute a promising molecular therapeutic target for head and neck tumors. Overall, these findings aim to emphasize the use of the chick embryo as an *in vivo* model for the screening of anticancer compounds.

## Summary

HNSCCs represent an aggressive class of neoplasms with a worldwide high incidence due to two main risk factors: tobacco/alcohol consumption and HPV infection.^32^ The translation of efficient therapeutic strategies in oncology requires a deep understanding beyond the physiopathology of neoplasms as well as ethical and reliable *in vivo* models that provide an analogous environment for an accurate exploration of the behavior of cancer tissues and their response to the therapeutic approaches. Due to its highly vascularized environment and immature immune responses, the CAM model provides an optimal environment for the grafting of cancer cell lines and patient-derived cancer cells.^33^

Here, we have presented a standardized and optimized protocol for the medium-/high-throughput production of solid tumors using the commercial HPV-negative head and neck cell line SCC-25. Each step of the protocol requires basic biological practice and experience. In general, the high survival rate of the embryos and tumor take rate are among the essential criteria considered for the efficiency of the protocol. Additionally, a detailed outline and explanation of the steps and further remarks have been included to provide a suitable guide for the generation of tumor grafts and subsequent imaging and biomolecular assays. The reported step-by-step method allows the establishment of a feasible *in vivo* model that can provide insights on the biological processes at the basis of oral malignancies and the development of new therapeutic strategies.^100^ The CAM model may push oncological research toward a more rapid evaluation and efficient screening/selection of conventional and emerging antitumor treatments.
